# Non-coding RNAs in the Pathogenesis of Multiple Sclerosis

**DOI:** 10.3389/fgene.2021.717922

**Published:** 2021-09-30

**Authors:** Aadil Yousuf, Abrar Qurashi

**Affiliations:** Department of Biotechnology, University of Kashmir, Srinagar, India

**Keywords:** multiple sclerosis, central nervous system, microRNA, long noncoding RNA, neurodegeneration

## Abstract

Multiple sclerosis (MS) is an early onset chronic neurological condition in adults characterized by inflammation, demyelination, gliosis, and axonal loss in the central nervous system. The pathological cause of MS is complex and includes both genetic and environmental factors. Non-protein-coding RNAs (ncRNAs), specifically miRNAs and lncRNAs, are important regulators of various biological processes. Over the past decade, many studies have investigated both miRNAs and lncRNAs in patients with MS. Since then, insightful knowledge has been gained in this field. Here, we review the role of miRNAs and lncRNAs in MS pathogenesis and discuss their implications for diagnosis and treatment.

## Introduction

Multiple sclerosis (MS) is a chronic inflammatory demyelinating neurodegenerative disease of the central nervous system (CNS) (Thompson et al., 2018). It mainly affects young adults, with onset between the ages of 20 and 40 years, and is predominant in women (Thompson et al., 2018). The pathological hallmark of MS is the accumulation of focal plaques, which are areas of demyelination along with infiltration of immune cells found throughout the CNS (Mahad et al., 2015).

The clinical manifestations and course of MS vary and are broadly divided into three types: relapsing-remitting MS (RRMS), primary progressive MS (PPMS), and secondary progressive MS (SPMS). Almost 85% of patients typically present with RRMS, which is characterized by episodes of disability, followed by a period of recovery (Confavreux and Vukusic, 2014; Mahad et al., 2015; Thompson et al., 2018). Approximately 10–15% of patients exhibit PPMS, which is characterized by a slow progression of disease from the beginning without remission (Confavreux and Vukusic, 2014; Thompson et al., 2018). Over 10 years, roughly half of RRMS patients progress to the SPMS stage characterized by chronic inflammation, sclerosis, and brain atrophy with few or no periods of remission (Confavreux and Vukusic, 2014; Thompson et al., 2018).

The pathophysiological mechanism of MS is heterogeneous and is thought to involve complex gene-environment interactions (Mahad et al., 2015). However, the major cause of MS development is a pro-inflammatory response. Immune cells such as CD4^+^ and CD8^+^ T cells, B cells, macrophages, and other cells infiltrate the CNS through a disrupted blood-brain barrier (BBB) (Mahad et al., 2015). These cells, together with resident activated microglia and astrocytes, damage oligodendrocytes and myelin through contact-dependent mechanisms and the secretion of cytokines and chemokines (Mahad et al., 2015).

In the initial stages of MS development, CD4^+^ T helper type 1 (Th1) and CD4^+^ T helper type 17 (Th17) are autoreactive to myelin and have therefore been intensively investigated (Sospedra and Martin, 2005). Both Th1 and Th17 cells are enhanced in the CNS, cerebrospinal fluid (CSF), and the blood of MS patients, as well as in the experimental autoimmune encephalomyelitis (EAE) model of MS. Th1 cells and Th17 cells produce cytokine interferon-*γ* (IFN-*γ*) and cytokine interleukin-17 (IL-17), respectively, which initiate inflammation and neuronal cell death (Sospedra and Martin, 2005). Inhibition of Th1 or Th17 cells or expansion of anti-inflammatory Th2 cells ameliorates disease in EAE animal models (Aharoni et al., 2000; O’Connor et al., 2008; Jäger et al., 2009). In addition, CD4^+^ regulatory T cells (Tregs), which normally prevent damage to host cells by limiting the immune response, are decreased in the frequency and suppressive function of MS (Sospedra and Martin, 2005). In EAE animal models, Treg cells and cytokine interleukin-10 (IL-10) negatively regulate disease development. In summary, the homeostasis of pro-inflammatory cells and anti-inflammatory and cytokine activities, including TNF-α, IFN-γ, IL-17, IL-6, and IL-18, appears to be significantly dysregulated in MS.

**FIGURE 1:**
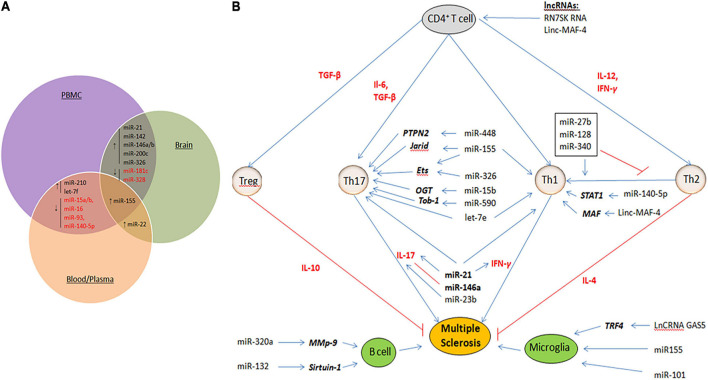
**(A)** Dysregulated miRNAs that are common in various samples (PBMC, blood/Plasma and brain tissue) of MS patients. ↑ represent up-regulated miRNAs and ↓ represent down-regulated miRNAs. **(B)** Mechanisms of ncRNAs in MS. Shown are the miRNAs and lncRNAs that function in the modulation of various immune cells, including upregulating activity of the proinflammatory Th1 cells and Th17 cells, B cells, and Microglia cells. Aberant expression of such ncRNAs eventually results in demyelination within the brain and spinal cord, and axonal damage. Cytokines that promote cell fate and differentiation are shown in red. Target genes of miRNAs are shown in bold italics.

Non-coding RNAs (ncRNAs) provide an intricate network that controls gene expression and immune system responses (Esteller, 2011; Wright and Bruford, 2011). They are tightly regulated and play critical roles in development and physiology. Therefore, their dysregulation plays an important role in the pathogenesis of MS. In this respect, microRNAs (miRNAs) and long non-coding RNAs (lncRNAs) have provided potential biomarkers and mechanistic insights into MS (Liu et al., 2014; Yang et al., 2018). miRNAs are small ncRNA molecules with lengths of 21–25 nucleotides that regulate gene expression at the post-transcriptional level by causing degradation or translational repression of target mRNAs (Qurashi et al., 2007). On the other hand, lncRNAs are a heterogeneous group of ncRNAs with a length of more than 200 nucleotides that regulate all steps of gene expression, including transcription, post-transcription, and translation (Mercer et al., 2009; Ponting et al., 2009; Chen and Carmichael, 2010; Hung and Chang, 2010; Ma et al., 2013; Beermann et al., 2016). In multiple studies, miRNAs and lncRNAs were profiled for expression in demyelinating lesions and body fluids [CSF, peripheral blood mononuclear cells (PBMCs), plasma, and whole blood] from MS patients (Yang et al., 2018; Ghaderian et al., 2020). These studies have identified a large set of miRNAs and lncRNAs that are dysregulated in MS. Some of these miRNAs or lncRNAs are dysregulated in a lineage-specific manner, in specific cell populations or during specific stages/subtypes of MS, providing new MS-specific biomarkers to predict disease progression or therapy response. Figure 1A summarizes some of the common miRNAs that have been identified as dysregulated in a variety of patient samples or studies. However, some critical issues that could lead to discrepancies in such studies need to be addressed. These include an appropriate sample number, biological sample (serum, plasma, PBMC, and blood), stage of the disease (RPMS, PPMS, and SPMS), and technical and analytical methods selected for analysis. Given the overwhelming number of ncRNAs identified in these studies, only a few have been functionally defined and are thus discussed here.

## miRNAs Involved in MS Pathogenesis

Multiple studies have reported a repertoire of aberrantly expressed miRNAs in both the immune and CNS cells of MS patients. Some of these miRNAs have been functionally characterized to play critical roles in MS pathogenesis. Table 1 summarizes the miRNAs and their targets, as described in the literature. While most of the miRNAs were found to be uniquely dysregulated in a particular cell type, some were dysregulated in many cell types and studies (Figures 1A,B).

**TABLE 1:** Dysregulated miRNAs in T cells, B cells, and monocytes of MS.

**Expressing cell**	**miRNA**	**Expression change**	**Targets**	**Functions**	**References**
CD4+T cells	miR-128, miR-27b, miR-340	Increased	BMI1, IL-4	Promote Th1 differentiation Inhibit Th2 differentiation	Guerau-de-Arellano et al., 2011
	miR27a			Inhibit negative regulators of Th17 cell differentiation	Ahmadian-Elmi et al., 2016
	let7e		IL-10	Promote Th17 differentiation	Guan et al., 2013
	miR-155		Est-1 and Jarid2 Dnaja2, Dnajb1	Promote Th17/Th1 differentiation Induces Th17 differentiation	O’Connell et al., 2010; Hu et al., 2013; Escobar et al., 2014; Zhang et al., 2014; Mycko et al., 2015; Vistbakka et al., 2017
	miR-15b		OGT	Inhibit Th17 differentiation	Liu R. et al., 2017
	miR-326		Ets-1	Stimulates Th17 differentiation	Du et al., 2009
	miR-20b	Decreased	RORγt STAT3	Inhibits the progression of EAE	Ingwersen et al., 2015
	miR-18a		CDC42	Decreases cells proliferation	Ingwersen et al., 2015
	miR-1405p		STAT1	Inhibits differentiation of Th1 cells	Guan et al., 2016
	miR-29a		TTP	Regulates of apoptosis	
	miR-103		KLF4	Promotes cell proliferation	
	miR-15a miR-16-1		BCL2 Cyclin D1 Cyclin D2 WT1, MCL1 YAP1, Sox5	Induces apoptosis	Lorenzi et al., 2012
	miR-590		Tob1	Differentiation of Th17	Liu Q. et al., 2017
	miR-448		IL-17 A, RORγt PTPN2	Stimulates Th17 differentiation	Wu et al., 2017
CD8^+^ T cells	miR- 629	Increased	TRIM33	Promotes TGFβ/Smad Signaling	Huang et al., 2016
CD4^+^ memory T cells	miR-29b		T-bet IFN-γ	Regulates Th1 cell proliferation	Smith et al., 2012
Naive T cells	miR-128		BMI1 GATA3	Inhibits MS development	Guerau-de-Arellano et al., 2011
Treg	miR-142-3p		Foxp3	Suppresses function of Treg cells	Arruda et al., 2015
	miR-27		c-Rel, FOXO1 RUNX1,SMAD2/3, IL-10, GZMB	Immunological tolerance	Cruz et al., 2017
	miR-25 miR-106b	Decreased	CDKN1 A/p21 BCL2L11/Bim	Regulates of TGF-β signaling pathway	De Santis et al., 2010
pre-B cells	miR-106b-25 cluster	Decreased	PTEN	Increases apoptosis	Sievers et al., 2012
			Bim	Suppresses pro-B to pre-B development	Ventura et al., 2008
	miR-17–92 clusters				
B cells	miR-320a		MMP-9	Damages to the BBB Enhances permeability of the barrier	Aung et al., 2015; Sun et al., 2015; Wang et al., 2015
	miR-132	Increased	Sirtuin-1 PRKAG3	Elevates expression of lymphotoxin and TNF-α Induces cell migration	Miyazaki et al., 2014
Monocytes	miR-17-5p miR-20a miR-106a miR-106a–92 families	Decreased	AML1	Stimulates the transcription of M-CSFR Causes the differentiation and maturation of monocytes	Fontana et al., 2007
Monocytes DC	miR-146a	Increased	TRAF6 IRAK1	Regulates of TLR signaling pathway	Jurkin et al., 2010

## Dysregulation of miRNA in the Immune Cells of Multiple Sclerosis Patients

The upregulated expression of miR-326 in Th17 cells isolated from the peripheral blood of MS patients with RRMS was related to disease severity and the production of IL-17 (Du et al., 2009). It inhibits Ets-1, a negative regulator of Th17 cell differentiation, and its overexpression increases Th17 cell number and leads to severe EAE. In contrast, miR-326 knockdown reduced the number of Th17 cells and alleviated EAE (Du et al., 2009). Similarly, miR-155 expression in T cells promotes Th17 differentiation and function by inhibiting the transcription of Ets-1 (O’Connell et al., 2010; Hu et al., 2013; Zhang et al., 2014). Similarly, miR-590 is upregulated in relapse-MS patients to promote the differentiation of Th17 cells by targeting Tob1 (Transducer of Erb-2), a member of the Tob/BTG anti-proliferative family of proteins (Liu Q. et al., 2017). In addition, miR-590 affects the pathogenicity of Th17 cells by upregulating inflammation-related molecules, such as CXCL3, CSF2, and IL-23R (Liu Q. et al., 2017). miR-448 can increase Th17 differentiation by directly inhibiting the anti-inflammatory protein tyrosine phosphatase non-receptor type 2 (PTPN2) (Wu et al., 2017). The let-7e miRNA was significantly upregulated in both experimental EAE and MS patients. Let-7e inhibition shifts the immune response to a Th2 profile and reduces disease severity, whereas let-7e overexpression increases Th1 and Th17 cells and worsens EAE (Guan et al., 2013).

Downregulation of many miRNAs has also been shown to influence Th1 or Th17 cell differentiation. For example, miR-15b targets O-linked N-acetylglucosamine transferase, which is normally required for CD4^+^ T cell activation and the ability to induce inflammation (Liu R. et al., 2017). However, in patients with MS, miR-15b is downregulated to inhibit Th1 or Th17 cell differentiation. In-line knockdown of miR-15b aggravated EAE, and overexpression of miR-15b alleviated EAE. Similarly, the miR-132 cluster is downregulated in CD4^+^ cells (Hanieh and Alzahrani, 2013). It targets Bcl-6 (B-cell lymphoma 6), a negative regulator of Th17 differentiation. Its downregulation is associated with the severity of EAE (Hanieh and Alzahrani, 2013). Similarly, under normal conditions, miR-1405p inhibits the differentiation of Th1 cells by downregulating STAT1 (signal transducer and activator of transcription) (Guan et al., 2016). However, its expression is markedly downregulated in MS, and consequently increases the development of Th1 cells and disease severity (Guan et al., 2016).

Dysregulation of miRNAs in MS is not limited to T helper cells, but has been reported in other types of T cells to influence their polarization. For example, miR-128 and miR-27b expression is elevated in naïve CD4^+^T cells, and miR-340 expression is increased in CD4^+^ memory T cells (Guerau-de-Arellano et al., 2011). These miRNAs inhibit the differentiation of Th2 cells by directly decreasing the expression of IL-4 and BMI1 (B lymphoma Mo-MLV insertion region 1 homolog), resulting in a Th2 to Th1 response shift. Interestingly, oligonucleotides against these miRNAs restore Th2 responses in patients with MS (Murugaiyan et al., 2011). In CD4^+^ memory T cells, miR-29b, which is induced by IFN-γ, acts in a negative feedback loop by inhibiting T-bet and IFN-γ transcription to control Th1 cell bias (Smith et al., 2012). Treg cells in MS have differential expression of 23 miRNAs when compared to healthy controls (De Santis et al., 2010). Among the significantly increased miRNAs were miR-106b and miR-25, which influenced TGF-β signaling, which is important for the development of both Th17 and Treg cells (Petrocca et al., 2008). TGF-β signaling is also increased in pro-inflammatory CD8^+^ T cells through the upregulation of miR-629 (Huang et al., 2016). Together, these lines of evidence have demonstrated that miRNAs through the inhibition of various target genes influence the differentiation of proinflammatory Th1 cells and Th17 cells, the development of Tregs, and the alteration of the Th2 to Th1 response in MS.

## Dysregulation of miRNAs in Antigen-Presenting Cells (B Cells, Macrophages, and Dendritic Cells) of Multiple Sclerosis Patients

In addition to T cells, several important miRNAs have been identified to be differentially expressed in B lymphocytes of MS (Table 1). The expression of miR-320a is considerably decreased in B cells (Aung et al., 2015). Among many targets, miR-320a inhibits matrix metallopeptidase-9 (MMP-9) produced by activated B cells. In MS, increased MMP-9 expression and secretion in B cells due to downregulation of miR-320a disrupts the BBB and degrades myelin basic protein (Chandler et al., 1995; Asahi et al., 2001). Similarly, significantly increased expression of miR-132 in patients with MS reduces the level of Sirtuin-1 in B lymphocytes, which in turn accounts for the elevated expression of pro-inflammatory cytokines such as lymphotoxin and tumor necrosis factor (TNF-α) (Miyazaki et al., 2014). miR-17–92 inhibits the expression of Bim, a pro-apoptotic protein, and PTEN, a tumor suppressor. Therefore, downregulation of miR-17–92 results in elevated levels of Bim, which in turn suppresses the development of pro-B to pre-B cells (Ventura et al., 2008). Compared to controls, in untreated RRMS patients, miR-155 expression was significantly increased in both peripheral circulating CD14+ monocytes and active lesions CD68+ cells from perivascular (blood-derived macrophages) and parenchymal (microglia) brain regions. Upregulation of miR-155 in these cells was subsequently associated with increased proinflammatory cytokine secretion (Moore et al., 2013). miR-124 is considered a key regulator of microglial quiescence. Accordingly, in EAE, miR-124 expression is decreased in activated microglia, while overexpression of miR-124 could promote activated microglia into a phenotype resembling microglia quiescence and suppress EAE by deactivating macrophages via the C/EBP-α-PU.1 pathway (Ponomarev et al., 2011). Together, these studies demonstrate the crucial effects of miRNAs on antigen-presenting cell-mediated mechanisms by influencing their activation and effector functions.

## Dysregulation of miRNA in CNS Cells of Multiple Sclerosis Patients

Similar to immune cells, aberrant expression of miRNAs in the CNS contributes to the mechanism underlying inflammation in MS. In a study examining cell type-specific miRNA profiles using laser capture microdissection, 10 miRNAs were identified to be substantially elevated in active MS lesions (Junker et al., 2009). CD47, which is ubiquitously expressed in a variety of human cells to prevent phagocytosis, was decreased in the active lesions of MS patients. Three miRNAs, miR-155, miR-34a, and miR-326, which are upregulated in MS, can target CD47, consequently releasing macrophages from inhibitory control and promoting myelin breakdown (Junker et al., 2009). These changes occur primarily in astrocytes. In addition, miR-155 directly targets and downregulates SOCS1, a negative regulator of cytokine production in astrocytes (Moore et al., 2013; Arruda et al., 2015). miR-155 also targets the neurosteroid synthesis enzymes ARK1C1 and ARK1C2 (Noorbakhsh et al., 2011). miR-23 control of lamin B1 was shown to be important for oligodendrocyte growth and myelination in a previous study, implying that it may play a role in the pathogenesis of MS (Lin and Fu, 2009). Compared to controls, miR-219 and miR-338 are reduced in patients with MS (Dugas et al., 2010). Overexpression of miR-219 and miR-338 promotes the differentiation of oligodendroglial precursor cells (OPCs) in culture. Milbreta et al. (2019) introduced these miRNAs into the oligodendrocytes of rats and demonstrated their therapeutic potential in promoting oligodendrocyte differentiation and myelination. Based on these findings, it is clear that dysregulation of miRNAs affects an environment that promotes remyelination and axon regeneration, both of which are impaired in MS.

## lncRNAs in MS

Although research into the role of lncRNAs in MS is still in its infancy, abnormal lncRNA expression has been investigated in serum, plasma, PBMCs, and blood exclusively in patients with RRMS and SPMS (Santoro et al., 2016). Like miRNA, lncRNAs can modulate the activity of various immune cells (Figure 1B). In MS patients, excess *NEAT1* leads to re-localization of SFPQ (splicing factor proline/glutamine-rich) from the IL-8 promoter, resulting in transcriptional activation of IL-8 (Imamura et al., 2014). *RN7SK RNA* is involved in the regulation of CD4^+^ T lymphocytes and contributes to inflammation (Sung and Rice, 2006). The upregulation of RN7SK RNA in the 7SK snRNP complex might catalytically repress P-TEFb, a Cdk9/cyclin T1 kinase complex, which is important for the differentiation of CD4^+^ T cells into several sub-populations. *TUG1* is upregulated in several neurodegenerative diseases, including MS. The TUG1 promoter contains conserved p53-binding and is a downstream target of p53 participating in the apoptotic pathways (Rossi et al., 2014). Sun et al. (2017) documented the upregulation of the lncRNA growth arrest-specific 5 (GAS5) in amoeboid-shaped microglial cells of MS patients. GAS5 was demonstrated to promote polarization of the M1 subgroup of microglial cells and, consequently, demyelination. Interfering with GAS5 in transplanted microglia slowed the course of EAE and promoted remyelination. GAS5 inhibits the proliferation of T cells by binding to PRC2, the polycarbonate 2 suppressor complex, and suppresses IRF4 transcription factor. Zhang et al. found that linc-MAF-4 levels were considerably higher in PBMCs from MS patients than in healthy controls. Linc-MAF-4 exacerbates MS pathogenesis by altering the Th1/Th2 ratio and by targeting MAF, a Th2 cell transcription factor required for Th2 differentiation (Zhang et al., 2017). Given these findings and the ability of lncRNAs to activate or repress gene expression at multiple levels through various mechanisms, it is conceivable that they act as critical regulators in the pathogenesis of MS. They are also promising biomarker candidates for MS diagnosis because of their stability in body fluids and cell specificity.

## Conclusion and Perspectives

Multiple sclerosis is a chronic inflammatory disease of the nervous system with autoimmune manifestations. The complexity associated with its pathophysiology and clinical presentation underlies the need for specific biomarkers and therapies. Large repertoires of aberrantly expressed miRNAs and lncRNAs have been identified in the immune and CNS cells of MS patients. Although the role of some of these miRNAs or lncRNAs in mediating MS pathogenesis has been demonstrated, others need to be functionally characterized. Interestingly, several miRNAs that are dysregulated in MS meet most of the required criteria for being an ideal biomarker, such as accessibility, high specificity, and sensitivity.

Given the wide range of cells and immunological responses implicated in MS pathogenesis, as well as the numerous targetsof ncRNAs (particularly miRNAs), it is important to explore specific target genes and pathways that drive aberrant immune responses in MS. Therefore, the combination of ncRNAs and their targets may provide better signatures for developing specific biomarkers and new therapeutic interventions in MS. In this context, disease-modifying therapies may include drugs and treatment methods that modulate ncRNA expression or function. Because some of the immune abnormalities in MS have been described in other types of autoimmune diseases, it is reasonable to assume that such studies will add to our understanding of the complex regulatory networks in autoimmune disorders in general.

## Author Contributions

Both authors listed have made a substantial, direct and intellectual contribution to the work, and approved it for publication.

## Conflict of Interest

The authors declare that the research was conducted in the absence of any commercial or financial relationships that could be construed as a potential conflict of interest.

## Publisher’s Note

All claims expressed in this article are solely those of the authors and do not necessarily represent those of their affiliated organizations, or those of the publisher, the editors and the reviewers. Any product that may be evaluated in this article, or claim that may be made by its manufacturer, is not guaranteed or endorsed by the publisher.
